# The effect of Dapagliflozin on renal functions in hospitalized patients with Acute Heart Failure

**DOI:** 10.12669/pjms.39.6.7525

**Published:** 2023

**Authors:** Syedah Fauzia Fatima Gilani, Shabana Ali, Muhammad Bilal Siddique, Kulsoom Farhat, Mudassar Noor, Farzana Waqar

**Affiliations:** 1 Syedah Fauzia Fatima Gilani, MBBS, MPhil, Pharmacology Department, Army Medical College, Rawalpindi, Pakistan; 2 Shabana Ali, MBBS, MPhil, FCPS, Pharmacology Department, Army Medical College, Rawalpindi, Pakistan; 3 Muhammad Bilal Siddique, MBBS, MCPS, FCPS Armed Forces Institute of Cardiology, Rawalpindi, Pakistan; 4 Kulsoom Farhat, MBBS, MPhil, PhD, Pharmacology Department, Army Medical College, Rawalpindi, Pakistan; 5 Mudassar Noor, MBBS, MPhil, PhD, Pharmacology Department, Army Medical College, Rawalpindi, Pakistan; 6 Farzana Waqar, Pharmacology Department, Army Medical College, Rawalpindi, Pakistan

**Keywords:** Dapagliflozin, Acute heart failure, Serum creatinine, eGFR

## Abstract

**Objective:**

To evaluate the influence of dapagliflozin on renal functions and diuretics use in patients with acute heart failure (AHF).

**Methods:**

This comparative analytical study was conducted at Armed Forces Institute of Cardiology, Rawalpindi from July 2022 to November 2022. Patients were distributed equally in two groups i.e. Dapagliflozin and Conventional Groups, where patients received dapagliflozin added to conventional therapy for AHF and, only conventional therapy for AHF respectively. Estimated glomerular filtration rate (eGFR), serum creatinine were measured and compared on admission, after 48 hours and on discharge. Weight loss during hospitalization, daily dose of furosemide and length of hospital stay was also recorded. Quantitative parameters were analyzed using t-test or Mann Whitney U test accordingly.

**Results:**

There were no significant baseline differences in renal functions. A modest decline in eGFR was observed in both groups after 48 hours. However, the variation in values of eGFR remained similar among both groups after 48 hours *(p*-value 0.365) and on discharge (*p*-value 0.768). Whereas, patients subjected to dapagliflozin treatment exhibited a more profound diuretic response expressed as greater weight loss (*p*-value < 0.001), achieved at comparatively lower doses of loop diuretics. Moreover, they also had a shorter duration of hospital stay (six vs eight days, *p*-value <0.001).

**Conclusion:**

Institution of dapagliflozin did not cause any significant deterioration of renal functions, whereas; it was associated with improved diuretic response as depicted by more pronounced weight loss at comparatively lower doses of loop diuretics.

## INTRODUCTION

Heart failure (HF) is the leading cause of morbidity and mortality. According to the latest estimations, globally 64 million people are suffering from HF and the prevalence is 1-2%. The existence of HF reaches up to 10% in age above 65 years.^1^ A quick emergence of de novo or precipitation of existing HF signs and symptoms are termed as acute heart failure (AHF). It is amongst the main reasons of unplanned hospitalizations and untoward outcomes in 65 and those aged more than 65.^1^ Around 25% of patients hospitalized with AHF are re-admitted within a month of discharge, mortality during this period extends up to 10%.^2^ Congestion, excess fluid and hemodynamic instability are the main reasons for urgent hospitalizations in AHF. Relief in congestion and fluid removal are the key therapeutic goals for the treatment of AHF. For decades loop diuretics are the mainstay for the management of AHF, specifically during hospital stay.

However, majority of patients are unable to achieve adequate decongestion because of the development of acute cardio-renal syndrome, characterized by an immediate decline in kidney function and the emergence of diuretic resistance.^3^ Moreover, the use of loop diuretics is also linked with the augmented adrenergic activity, and raised serum uric acid levels that may also lead to untoward results in patients with AHF.^4^ Multiple clinical researches, testing new pharmacotherapies in patients with AHF remained unsuccessful to demonstrate satisfactory results on the outcomes after discharge. The addition of vasopressin antagonists^5^,^6^, natriuretic peptides, or ultrafiltration to general diuretic treatments could not demonstrate desirable clinical benefits in patients with AHF.

Therefore, the scope for development of new and advanced approaches with the potential to improve outcomes in AHF is still open. A novel group of antidiabetic drugs that constitutes the sodium glucose transporter 2 (SGLT2) inhibitors have displayed encouraging results in patients with chronic heart failure (CHF) having diabetes mellitus Type-2 (T2DM). A remarkable decline was observed in heart failure hospitalizations (HFH) and mortality in CHF.^7^ A randomized clinical trial on dapagliflozin showed substantial decrease in HF events after introduction of the drug in CHF patients having reduced ejection fraction (EF) irrespective of their diabetic status.^8^ Despite favorable results in CHF, the efficiency of SGLT2 inhibitors in AHF still needs to be investigated.

During hospital admission fluctuation in renal functions is the prime concern. The patients admitted with AHF are in reception of high doses of loop diuretics and are at high end to develop worsening of renal function (WRF). Therefore, the agents that influence kidney function may have a distinctive impact on alterations in estimated glomerular filtration rate (eGFR) during hospitalization with AHF. The effect of SGLT2 inhibitors on renal functions during AHF have not been fully explored. This study aims to furnish the influence of Dapagliflozin on renal functions and diuretic use in patients with AHF during early phase of treatment.

## METHODS

This clinical research was conducted from July 2022 to November 2022 in Armed Force institute of Cardiology, Rawalpindi. Sample size of 64 was calculated based on the prevalence of HF^9^ by using Rao soft sample size calculator. However, sample size was increased to 160, enrolling 80 individuals in each group. Informed consent was taken before enrollment of the patients.

### Ethical Approval

The study was approved by Ethical Review Committee of Army Medical College, National University of Medical Sciences Rawalpindi (ERC/ID/205) and from Institutional Ethical and Review Board of the hospital (S/017/2022).

Eligible patients were enrolled via non-probability convenience sampling and were distributed equally in two groups on the basis of medication they received. One group received dapagliflozin 10 mg orally daily, as add on therapy to the conventional diuretic therapy for AHF. Dapagliflozin was started within 24 hours of admission and was maintained till the course of study. The other group was in reception of only conventional diuretic therapy for AHF. The conventional therapy includes commencement of intravenous (IV) furosemide in continuous infusion or in equally divided bolus doses.

The furosemide was administered at doses adequate to obtain optimal volume status and congestion relief. The suitable dose of furosemide was decided by attending physician. The average dose of furosemide initiated to all patients ranged from 60 to 120mg/24 hours. Change in dose of furosemide was attributed to the relief of congestion and its dose given per day was recorded. Laboratory data, specifically serum creatinine was collected at baseline, after 48 hours and on discharge. The subtleties regarding body weight were assessed as an indicator of adequate diuresis. Patient’s body weight was noted at the time of hospital admission and discharge. The length of hospital stay of all patients was also noted. The decision to discharge was made by the attending physician credited to improvement in clinical symptoms.

As per inclusion criteria, patients over 30 years of age both male and female hospitalized with AHF and requiring IV administration of furosemide were included. Diagnosis was made according to presenting signs and symptoms described by European Society of Cardiology^10^ which are dyspnea at rest or with nominal exertion, orthopnea, pulmonary congestion (crackles on chest auscultation), pedal edema, rapid weight gain, and ascites. Patients having EF<40% were included in the study irrespective of their diabetic status. Patients requiring mechanical ventilation, IV inotropes or vasopressors or presenting with cardiogenic shock, diabetes mellitus Type-1, and urinary tract infection were excluded. Patients already taking SGLT2 inhibitors or having allergy to SGLT2 inhibitors were not included. Patients having eGFR<30 mL/min/1.73m^2^, history of AHF primarily prompted by acute myocardial infarction and pregnant women were not included.

The main outcome was WRF described as an increase in serum creatinine level of 0.3 mg/dL or more within 48 hours (Kidney Disease: Improving Global Outcomes (KDIGO) criteria).^11^ Chronic Kidney Disease Epidemiology Collaboration (CKD-EPI) equation was employed to calculate eGFR by using serum creatinine levels.^12^ Secondary outcomes included weight loss during hospitalization, dose of furosemide, and length of hospital stay.

Shapiro Wilks test was applied to check normality of data. Student’s t-test and Mann-Whitney U test were employed for comparison among the groups. Paired t-test or Wilcoxon rank test for comparison within the groups. Chi Square test was applied to analyze categorical data. Stratification of data was done to determine the effect of confounding factors and was found to have insignificant effect on outcomes. Normally distributed continuous variables are presented as mean with standard deviation, and non-normally distributed variables as median and interquartile range. Confidence Interval 95% was used, and the level of significance was set at *p* ≤ 0.05. SPSS V.26 was used for statistical analysis.

## RESULTS

The participants in both groups were analyzed based on demographic and clinical characteristics and statistically insignificant results were demonstrated among both groups as shown in Table-I. There were no significant baseline differences in eGFR and serum creatinine. A modest and significant decline in eGFR was observed after 48 hours in both cohorts (Table-II, Table-III and Fig.1a). However, this change was statistically non-significant when compared between the groups (*p* >0.05). The values of eGFR and serum creatinine obtained on baseline, after 48 hours and on discharge showed statistically insignificant differences among the groups (*p*>0.05) (Table-II, Fig.1a,1b).

**Table-I:** Baseline characteristics.

Variables	Dapagliflozin Group	Conventional Group	p -value
Age (years)	64.51±9.07	67.03±8.33	0.069
Male	67 (83.75)	61 (76.25)	0.236
Female	13 (16.25)	19 (23.75)	
BMI (kg/m^2^)	23.97±2.95	24.6±2.97	0.160
Hypertension	39 (48.75)	43 (53.75)	0.527
Dilated cardiomyopathy	33 (41.25)	34 (42.50)	0.873
Ischemic Heart Disease	41 (51.25)	36 (45)	0.429
Atrial Fibrillation	12 (15)	11 (13.75)	0.822
Diabetes Mellitus	40 (50)	40 (50)	----
NYHA Class III	45 (56.25)	43 (53.75)	0.751
NYHA Class IV	35 (43.75)	39 (48.75)	0.526
Systolic BP (mm Hg)	123.82±14.73	125.56±13.69	0.441
Diastolic BP (mm Hg)	76.76±10.38	75.60±9.81	0.468
HR beats /min	80 (74.25-84)	80.5 (75.25-84.75)	0.933
Hemoglobin mg/dL	12.15±1.95	12.01±1.94	0.642
Platelets Count	226.07±83.80	215.62±66.2	0.383
Random Blood glucose mg/dL	159 (109-223)	146 (100.5-212)	0.556
LVEF%	30 (25-35)	30 (25-35)	0.527
***Medication***			
Diuretics	80 (100)	80 (100)	------
Beta Blockers	62 (77.50)	65 (81.25)	0.558
ACEI/ARB/ARNI	56 (70)	50 (62.50)	0.316
Values are presented as n(%), mean ± SD, or median IQR			

BMI:Body mass index. BP:Blood Pressure, HR:Heart Rate, LVEF:Left ventricular ejection fraction, IQR:Interquartile range ACEI:Angiotensin Converting Enzyme inhibitor, ARB:Angiotensin receptor blocker, ARNI:Angiotensin receptor-Neprilysin inhibitor.

**Table-II:** Variations in Renal Functions.

Variable	Dapagliflozin Group	Conventional Group	p -value

eGFR mL/min/1.73m^2^ Median (IQR)			
Baseline	59 ( 49-75.10)	54.95 ( 47.5-71.75)	0.237
After 48hours	52.35(44-64.30)	49.10 (43.8-59.67)	0.365
Discharge	58.60 (47.3-72.05)	57.50 (48.85-68.52)	0.768

*Serum Creatinine mg/dL Mean ± SD*			

Baseline	1.24± 0.308	1.27± 0.328	0.464
After 48 hours	1.37± 0.320	1.47± 0.380	0.071
Discharge	1.28± 0.304	1.3 ±0.297	0.698

**Table-III:** Variation in renal functions within groups.

Variable	Dapagliflozin group		p- value	Conventional group		p- value
	
	Baseline	After 48 hours		Baseline	After 48 hours	
eGFR mL/min	59 ( 49-75.10)	52.35(44-64.30)	0.001	54.95 ( 47.5-71.75)	49.10 (43.8-59.67)	0.002
Serum Creatinine mg/dL	1.24± 0.308	1.37± 0.320	0.001	1.27± 0.328	1.47± 0.380	0.001
	*Baseline*	*Discharge*		*Baseline*	*Discharge*	
eGFR mL/min	59 ( 49-75.10)	58.60 (47.3-72.05)	0.202	54.95 ( 47.5-71.75)	57.50 (48.85-68.52)	0.953
Serum creatinine mg/dL	1.24± 0.308	1.28± 0.304	0.150	1.27± 0.328	1.3 ±0.297	0.391

**Fig.1:**
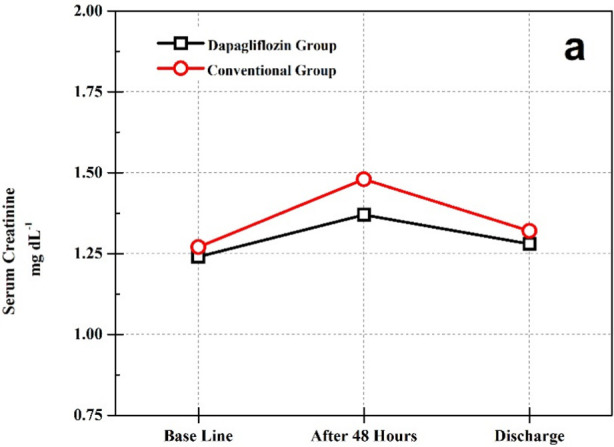
(a) Variation in Serum creatinine levels over time.

**Fig.1:**
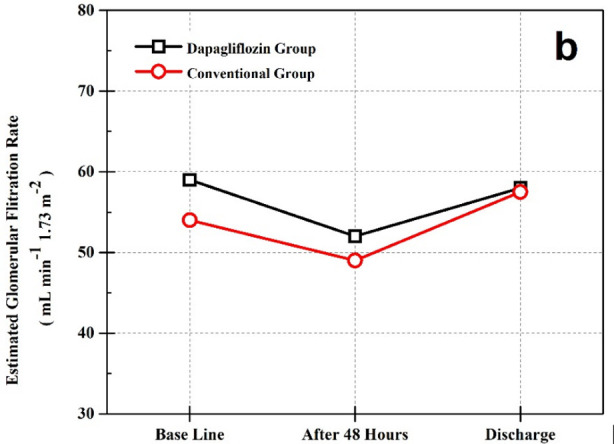
(b) Variation in eGFR over time.

When compared within the groups a similar pattern of fluctuations in renal functions was observed in both cohorts. A significant decline in eGFR was demonstrated by both cohorts that was no longer apparent at the time of discharge (Table-III). When the average doses of furosemide were compared a significant difference was revealed. The need to increase the dose of furosemide occurred less frequently in the dapagliflozin group. Patients subjected to dapagliflozin depicted greater weight loss during hospitalization and had shorter length of hospital stay (Table-IV).

**Table-IV:** Outcomes of Hospitalization.

VARIABLE	Dapagliflozin Group	Conventional Group	p -value
Weight Loss during Hospitalization(kg) (Mean ±SD)	3.7 ± 0.63	2.4 ± 0.73	< 0.001
Dose of Furosemide/day (mg) (Median IQR)	80 (60-120)	120 (90-120)	< 0.001
Length of Hospital Stay(days) (Median IQR)	6 (5-7)	8 (6-9)	< 0.001

## DISCUSSION

In this study we have explored the efficacy of SGLT2 inhibitor, 10 mg dapagliflozin in AHF during hospital admission as add on therapy. Evaluation of renal functions in our study demonstrated that the initiation of dapagliflozin in AHF is not associated with noteworthy deterioration of renal functions. An acute decline in eGFR within 48 hours was observed in both groups that was no longer apparent at the time of discharge. Decline in eGFR remained statistically insignificant when compared between the groups and the variation in renal functions observed during the course of study was similar among both study groups. Whereas our findings indicate an improved diuretic response in the dapagliflozin group, depicted by greater weight loss, with a tendency to use lower doses of furosemide as compare to the Conventional group.

In AHF, an aggressive approach for thorough decongestion is encouraged which may cause a decline in eGFR that is usually a transient occurrence in such case.^11^ GFR is the most reliable clinical indicator to measure renal functions as it emulates the functioning of nephrons.^12^ An acute but reversible decline in eGFR referred as GFR ‘dip’ has been linked to the usage of SGLT2 inhibitors in CHF.^13^ Keeping this in consideration we evaluated eGFR in our study and demonstrated that a similar trend of transient rebuff was observed by both groups which was insignificant when compared between groups. The findings in our study coincides with Tamaki et al.^14^ who assessed the impact of SGLT2 inhibitor empagliflozin on eGFR in AHF with T2DM. They studied 60 patients and instituted empagliflozin added to conventional treatment in 30 patients. They also reported a transient dip in eGFR, which was statistically insignificant and comparable between the study groups. Tamaki et al. in their study also compared the role of empagliflozin on change in weight and, contrary to our observations, displayed statistically insignificant results with no remarkable change in weight after seven days.^14^

Recently, the EMPULSE^15^ trial also explored the effect of SGLT2 inhibitor in hospitalized patients with AHF and evaluated the impact of empagliflozin 10 mg on eGFR and diuretic use. In agreement with our observations, the trial demonstrated a remarkable net weight loss in empagliflozin group as compared to placebo significantly decreasing the dose of diuretics. Moreover, an early meek decline in kidney function that disappeared after 30 days, was detected in the trial. A decrease in dose of furosemide with dapagliflozin in AHF was also reported by Ibrahim et al.^16^ They also observed noteworthy change in body weight following initiation of dapagliflozin at comparatively lower doses of furosemide, in admitted patients with AHF having T2DM. Ibrahim et al. also compared serum creatinine levels between the two groups and demonstrated statistically insignificant findings.

Dapagliflozin exhibit an inhibiting effect on the SGLT2 receptors in kidneys. Inhibition of SGLT2 receptors leads to increased excretion of plasma glucose initiating osmotic diuresis resulting in significant reduction in intravascular fluid.^17^ In our work, we assessed the diuretic response and inferred it by any change in body weight. Although it is a crude way for assessment of diuresis, yet it is convenient and has been adopted in numerous studies.^18^ Diuresis allied weight reductions mirrors fluid loss from the interstitial compartment.

Majority of AHF patients develop diuretic resistance and it becomes difficult to improve symptomatology with traditional loop diuretics. Management of such patients becomes tricky and may require addition of some other pharmacological agent.^19^ Recently there has been growing interest in exploring the effect of SGLT2 inhibitors in AHF. Multiple references from literature prefers 10 mg dapagliflozin as an optimal recommendation for HF patients.^7^

Our study population included both diabetic and nondiabetic patients. Dapagliflozin, despite being an antidiabetic drug, was found to be effective in nondiabetic patients as well. Nevertheless, addition of another agent with high doses of loop diuretics comes with its own risks. However, our findings support the early institution of dapagliflozin in AHF. In comparison to conventional group an improved diuretic response was observed in dapagliflozin group depicted by greater weight loss, without causing any significant deterioration of the renal functions. Multiple references from literature support and further prove the findings of our study. Therefore, dapagliflozin can be recommended as add on therapy in AHF.

### Limitations

This was a single-center study and included 160 patients. A multicenter approach with a larger sample size may provide more precise results.

## CONCLUSION

Our results supports SGLT2 inhibitors therapy during AHF hospitalization. The institution of dapagliflozin as add on therapy to the conventional diuretic therapy for AHF did not result in noteworthy WRF. Whereas, it demonstrated a better diuretic response depicted by more apparent weight loss with lesser use of loop diuretics.

### Authors’ Contribution:

**SFFG, SA**: Conceived, designed, collected data and did statistical analysis with interpretation & manuscript writing.

**MBS, FW**: Supervised the entire project in the hospital.

**SA, MN & KF**: Did review and final approval of manuscript, responsible for accuracy and integrity of data.
